# Survival Following Segmentectomy or Lobectomy in Patients With Stage IB Non-small-cell Lung Cancer

**DOI:** 10.3389/fonc.2020.00661

**Published:** 2020-05-15

**Authors:** Bo Hao, Lin Zhang, Tao Fan, Bohao Liu, Wenyang Jiang, Hao Hu, Qing Geng

**Affiliations:** Department of Thoracic Surgery, Renmin Hospital of Wuhan University, Wuhan, China

**Keywords:** non-small-cell lung cancer, segmentectomy, lobectomy, overall survival, lung cancer-specific survival

## Abstract

**Background:** Lobectomy with mediastinal lymph node dissection has always been recognized as the standardized treatment for early-stage non-small-cell lung cancer. However, the feasibility of segmentectomy performed in stage IB non-small-cell lung cancer (NSCLC) patients remains controversial. The present study aims to investigate whether the outcome of stage IB NSCLC patients undergoing segmentectomy was comparable to those who underwent lobectomy.

**Method:** We retrospectively collected data of 11,010 patients with primary stage IB non-small-cell lung cancer from the Surveillance, Epidemiology, and End Results database. Overall survival (OS) and lung cancer-specific survival (LCSS) were assessed among patients who were performed lobectomy or segmentectomy. To further assess the impact of the surgical procedures on patients with different tumor sizes, subgroups stratified by tumor size were analyzed.

**Results:** A total of 11,010 patients who were pathologically confirmed to be stage IB were included, of whom 10,453 received lobectomy and 557 received segmentectomy. Both univariate and multivariate Cox regression analyses showed that the patients receiving lobectomy had better OS [hazards ratio (HR) = 1.197, 95% confidence interval (CI) (1.066, 1.343), *P* < 0.001] than those receiving segmentectomy. However, multivariate analysis showed that there was no significant difference in LCSS between lobectomy and segmentectomy [HR = 1.172, 95% CI (0.963, 1.427), *P* = 0.114]. Meanwhile, subgroup analyses showed that lobectomy rather than segmentectomy was associated with better OS [HR = 1.278, 95% CI (1.075, 1.520) *P* = 0.006] and LCSS [HR = 1.118, 95% CI (1.005, 1.280), *P* = 0.047] for patients with a tumor size (TS) of ≤ 40 and >30 mm, while for patients with a TS of ≤ 30 mm, lobectomy yielded similar OS [TS ≤ 20 mm: HR = 1.068, 95% CI (0.853, 1.336), *P* = 0.566; TS > 20 mm and ≤ 30 mm: HR = 1.195, 95% CI (0.961, 1.487), *P* = 0.109] and LCSS [TS ≤ 20 mm: HR = 1.029, 95% CI: (0.682, 1.552), *P* = 0.893; TS > 20 and ≤ 30 mm: HR = 1.144, 95% CI (0.795, 1.645), *P* = 0.469] to that of segmentectomy.

**Conclusion:** Segmentectomy achieved equivalent OS and LCSS in stage IB NSCLC patients with TS ≤ 30 mm compared with lobectomy. Lobectomy showed better OS and LCSS than segmentectomy for patients with a TS of >30 and ≤ 40 mm. Segmentectomy may be acceptable in patients with an older age and a smaller TS.

## Introduction

Lung cancer has been the leading cause of cancer mortality worldwide, which makes lung cancer a major public health problem in the world (1). It is estimated that 234,030 cases were newly diagnosed and 154,050 died per year in the United States (2). According to the statistics, non-small-cell lung cancer (NSCLC) accounts for ~80% of all lung cancer cases (3). Thus, better treatment for NSCLC is urgently needed.

A study based on the Surveillance, Epidemiology, and End Results (SEER) database showed that NSCLC patients with a tumor size (TS) ≤ 1 cm, who underwent segmentectomy, had equivalent overall survival (OS) compared to those who had lobectomy (4). Later, an observational study using the same database demonstrated that lobectomy yielded better survival than segmentectomy in NSCLC patients with TS ≤ 2 cm who were diagnosed between 2000 and 2012 (5). Moon et al. (6) demonstrated that there were no significant differences in OS or lung cancer-specific survival (LCSS) among patients with TS ≤ 2 cm who underwent lobectomy vs. segmentectomy. Recently, a meta-analysis by Jsseldijk et al. (7) suggested that OS and disease-free survival (DFS) after segmentectomy yielded equal survival compared to lobectomy in NSCLC patients with stage T1aN0M0.

Thus, more and more studies suggest that segmentectomy yielded an equivalent survival rate compared to lobectomy in early-stage NSCLC patients. However, its survival comparison with lobectomy in stage IB non-small-cell lung cancer patients remains unknown. The present study aims to evaluate the impact of lobectomy and segmentectomy on OS and LCSS in stage IB (T2aN0M0) NSCLC patients using the SEER database.

## Methods

### Data

Clinical information of all patients was obtained from the SEER database, which was supported by the National Cancer Institute. The database aims to collect and report the cancer incidence and survival data from several registries that involve more than 30% of the U.S. population and has been used for survival analyses in numerous high-quality studies (5, 8–12).

### Study Population

The inclusion of the patients involved in the study include (1) NSCLC confirmed by pathology; (2) T2aN0M0 stage tumor based on the eighth edition of NSCLC stage classification (TS > 30 and ≤ 40 mm or TS ≤ 30 mm but involved with visceral pleural invasion); and (3) surgical history of lobectomy (surgery code: 30 and 33, and extended lobectomy was excluded) or segmentectomy (surgery code: 22, and wedge resection was excluded). And the exclusion criteria include (1) history of chemotherapy; (2) history of radiotherapy; (3) pathologically confirmed small cell lung cancer or all subtypes of sarcoma; (4) age <18; (5) tumor located in the main bronchus, as a result of which segmentectomy was impossible to be performed.

The baseline characteristics of patients were obtained from the datasets: age, gender, race, year of diagnosis, location of tumor, laterality, pathology classification, the number of resected lymph nodes, TS, survival status, survival time, and cause of death. All eligible patients were divided into the segmentectomy group or lobectomy group according to the surgical strategies. History of malignancy was categorized as No (having no other malignancies before lung cancer diagnosis) and Yes (having one or more malignancies before lung cancer diagnosis). Grade well/moderate group included grade I and II, while poor/undifferentiated included III and IV.

The primary endpoint of this analysis was OS, which is calculated from the day of surgery to the last follow-up or death. The secondary endpoint was LCSS, calculated from the day of surgery to the day of NSCLC-related death or the date of the last follow-up.

### Statistical Analysis

Conventional statistics are used to summarize the characteristics of the study. The Wilcoxon tests were used to calculate the distributions of continuous data (age, number of resected regional lymph nodes, and TS), and the Pearson χ^2^ test was used in categorical variables (sex, location, laterality, pathology, grade, and history of malignancy). Survival curves of OS and LCSS were calculated by the Kaplan–Meier method, and the significance was assessed by the log-rank test. To evaluate the impact of segmentectomy or lobectomy on the outcome of the patients, univariate and multivariate Cox regression analyses were used to calculate hazards ratios (HR) and 95% confidence intervals (95% CI).

SPSS 22.0 software (IBM Corporation, Armonk, NY, USA) was used for statistical analysis. Kaplan–Meier survival curves were established using R 3.6.1 (R Development Core Team, R Foundation for Statistical Computing, Vienna, Austria). All statistical tests were two-sided, and *P* < 0.05 was considered statistically significant for all statistical tests.

## Results

### Characteristics of the Study

A total of 11,010 NSCLC patients who were pathologically confirmed to be stage T2aN0M0 were included, of whom 10,453 underwent lobectomy and 557 segmentectomy. The year of diagnosis spanned from 2004 to 2013. The age of the cohort ranged from 22 to 94, and the average was 69. To further explore the impact of age on survival, the cohort was divided into three groups by age: ≤ 60, 61–75, and >75. The median and mean follow-up times of the entire cohort were 56 and 116.37 months, respectively. Baseline characteristics were depicted in Table 1. As shown in Table 1, segmentectomy is more likely to be performed in patients with an older age, White race, left lung lesions, advanced tumor grade, and a smaller TS. And a smaller number of lymph nodes were likely to be resected in patients who underwent segmentectomy.

**Table 1:** Baseline characteristics of the study population.

	**Lobectomy (*n* = 10,453)**	**Segmentectomy (*n* = 557)**	***P*-value**
**Age**			<0.001
≤ 60	1,839 (17.6%)	63 (11.3%)	
61–75	5,540 (53.0%)	282 (50.6%)	
>75	3,074 (29.4%)	212 (38.1%)	
**Sex**			0.251
Male	5,084 (48.6%)	257 (46.1%)	
Female	5,369 (51.4%)	300 (53.9%)	
**Location**			<0.001
Upper	6,251 (58.9%)	336 (59.9%)	
Lower	3,359 (33.3%)	206 (37.4%)	
Others	843 (7.8%)	15 (2.7%)	
**Race**			0.805
White	8,883 (85.3%)	479 (86.2%)	
Black	831 (8.0%)	41 (7.1%)	
Others	739 (6.7%)	37 (6.8%)	
**Pathology**			0.400
Adenocarcinoma	6,539 (62.6%)	341 (61.2%)	
Squamous cell carcinoma	2,704 (25.8%)	141 (25.3%)	
Others	1,210 (11.6%)	75 (13.5%)	
**Laterality**			<0.001
Left	4,272 (40.9%)	313 (56.2%)	
Right	6,181 (59.1%)	244 (43.8%)	
**Grade**			0.004
Well/moderate	6,310 (60.4%)	297 (53.3%)	
Poor/Undifferentiated	3,560 (34.1%)	223 (40.0%)	
Unknown	583 (5.5%)	37 (6.7%)	
**No. of resected lymph nodes**			<0.001
0	322 (3.1%)	139 (25.0%)	
1–3	1,716 (16.4%)	172 (30.9%)	
≥4	7,931 (75.9%)	214 (38.4%)	
Unknown	484 (4.6%)	32 (5.7%)	
**Tumor size (mm)**			<0.001
≤ 20	2,230 (21.3%)	191 (34.3%)	
21–30	2,564 (24.5%)	157 (28.2%)	
31–40	5,659 (54.1%)	209 (37.5%)	
**History of malignancy**			<0.001
No	5,993 (57.3%)	256 (46.0%)	
Yes	4,460 (42.7%)	301 (54.0%)	

### Survival Analysis

**Figure 1:**
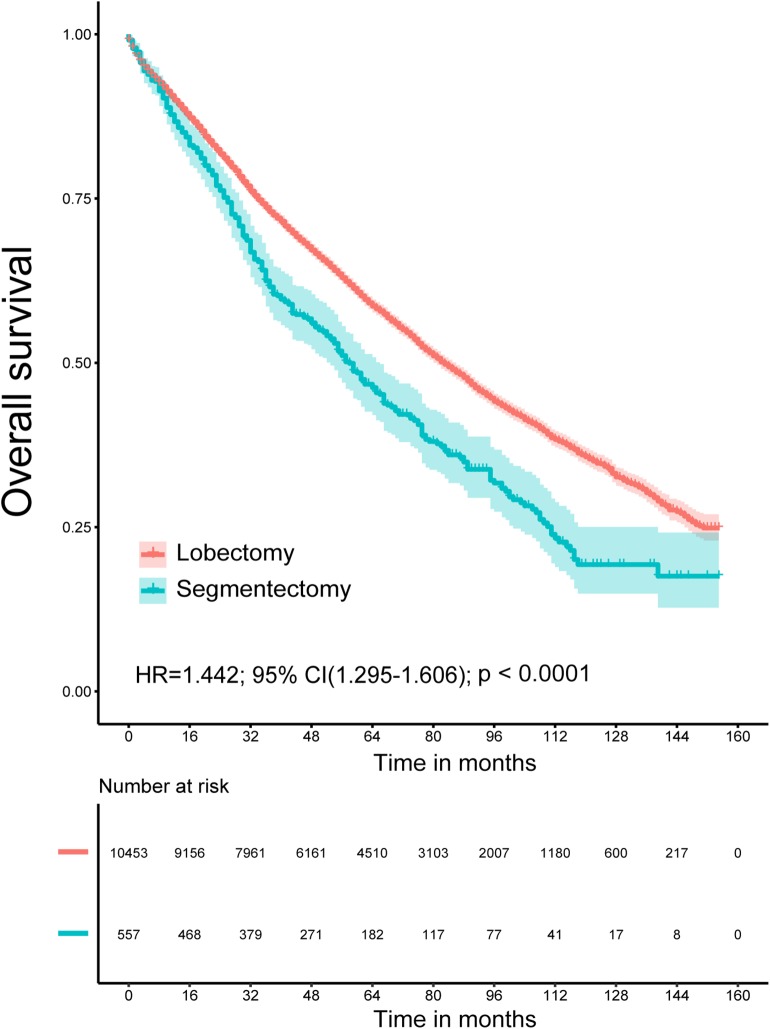
Overall survival for patients with lobectomy and segmentectomy.

**Figure 2:**
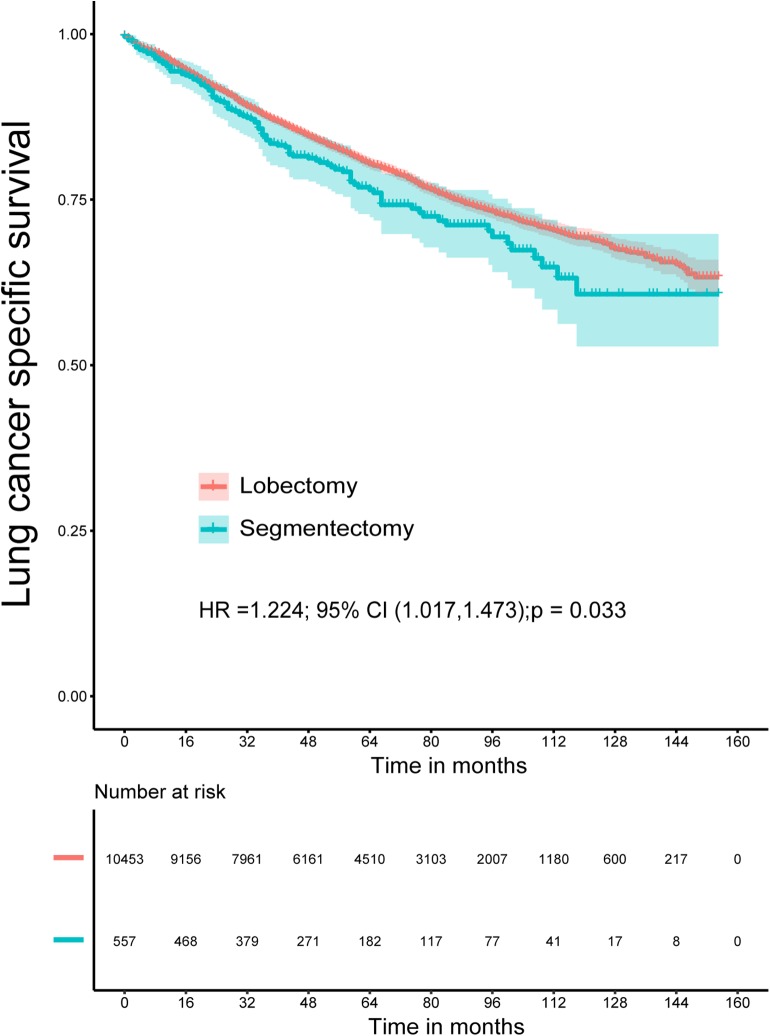
Lung cancer-specific survival for patients with lobectomy and segmentectomy.

The Kaplan–Meier survival analysis showed that lobectomy had better OS (*P* < 0.0001) and LCSS (*P* = 0.032) than segmentectomy (Figures 1, 2). And univariate and multivariate Cox regression analyses were further conducted. As shown in Tables 2, 3, univariate analyses suggested that an older age, male sex, White race, squamous cell carcinoma, advanced tumor grade, a smaller number of resected lymph nodes, and a larger TS yielded worse OS and LCSS. Patients undergoing segmentectomy had worse OS [HR = 1.442, 95% CI (1.295, 1.606), *P* < 0.001] and LCSS [HR = 1.224, 95% CI: (1.017, 1.473), *P* = 0.033]. In multivariate Cox regression, segmentectomy had poorer OS [HR = 1.197, 95% CI (1.066, 1.343), *P* = 0.002], but not poorer LCSS [HR = 1.172, 95% CI (0.963, 1.427), *P* = 0.114]. An older age, male sex, squamous cell carcinoma, advanced grade, and a smaller number of resected lymph nodes were associated with worse OS and LCSS. Interestingly, patients with a history of malignancy were associated with a better LCSS [HR = 0.311, 95% CI (0.280, 0.345), <0.001] compared with those without a history of malignancy; however, no significant difference was observed in OS [HR = 1.020, 95% CI (0.982, 1.075), *P* = 0.465].

**Table 2:** Univariate and multivariate regression analyses for overall survival.

	**Univariate analysis**			**Multivariate analysis**		
	**HR**	**95% CI**	***P***	**HR**	**95%CI**	***P***
**Age**						
≤ 60	1			1		
61–75	1.628	1.496–1.773	<0.001*	1.559	1.430–1.699	<0.001*
>75	1.777	1.684–1.875	<0.001*	2.420	2.213–2.647	<0.001*
**Sex**						
Male	1			1		
Female	0.703	0.667–0.740	<0.001*	0.707	0.669–0.746	<0.001*
**Race**						
White	1			1		
Black	0.856	0.773–0.947	0.003*	0.929	0.838–1.029	0.159
Others	0.771	0.690–0.861	<0.001*	0.845	0.756–0.945	0.001*
**Laterality**						
Left	1			1		
Right	0.991	0.941–1.045	0.748	1.038	0.983–1.096	0.179
**Location**						
Upper	1			1		
Lower	1.097	1.037–1.160	0.001*	1.067	1.008–1.128	0.025*
Others	0.978	0.884–1.083	0.673	0.956	0.861–1.062	0.402
**Pathology**						
Adenocarcinoma	1			1		
Squamous cell carcinoma	1.563	1.475–1.656	<0.001*	1.331	1.252–1.415	<0.001*
Others	1.273	1.174–1.381	<0.001*	1.168	1.074–1.270	<0.001*
**Grade**						
Well/Moderate	1			1		
Poor/Undifferentiated	1.277	1.210–1.348	<0.001*	1.177	1.112–1.246	<0.001*
Unknown	0.846	0.748–0.956	0.007*	0.863	0.763–0.977	0.012*
**Resected lymph nodes**						
0	1			1		
1–3	0.754	0.666–0.854	<0.001*	0.775	0.682–0.882	<0.001*
≥4	0.597	0.532–0.669	<0.001*	0.625	0.554–0.705	<0.001*
Unknown	0.645	0.549–0.757	<0.001*	0.680	0.577–0.802	<0.001*
**Tumor size (mm)**						
≤ 20	1					
21–30	1.272	1.176–1.376	<0.001*	1.180	1.090–1.276	<0.001*
31–40	1.314	1.227–1.407	<0.001*	1.159	1.081–1.243	<0.001*
**History of malignancy**						
No	1			1		
Yes	1.126	1.069–1.186	<0.001*	1.020	0.968–1.075	0.465
**Surgical procedure**						
Lobectomy	1					
Segmentectomy	1.442	1.295–1.606	<0.001*	1.197	1.066–1.343	0.002*

* *indicates that the difference was statistically significant*.

**Table 3:** Univariate and multivariate regression analyses for lung cancer specific survival.

	**Univariate analysis**			**Multivariate analysis**		
	**HR**	**95%C**	***P***	**HR**	**95%CI**	***P***
**Age**						
≤ 60	1			1		
61–75	1.243	1.103–1.402	<0.001*	1.394	1.234–1.574	<0.001*
>75	1.482	1.302–1.687	<0.001*	1.669	1.462–1.905	<0.001*
**Sex**						
Male	1			1		
Female	0.787	0.724–0.856	<0.001*	0.768	0.703–0.838	<0.001*
**Race**						
White	1			1		
Black	0.835	0.707–0.986	0.034*	0.847	0.716–1.003	0.054
Others	1.054	0.901–1.232	0.513	0.989	0.846–1.158	0.895
**Laterality**						
Left	1			1		
Right	1.060	0.973–1.154	0.181	1.099	1.007–1.199	0.035*
**Location**						
Upper	1			1		
Lower	1.050	0.959–1.149	0.290	1.064	0.972–1.166	0.180
Others	0.919	0.778–1.085	0.318	0.911	0.768–1.081	0.287
**Pathology**						
Adenocarcinoma	1			1		
Squamous cell carcinoma	1.407	1.279–1.548	<0.001*	1.222	1.105–1.351	<0.001*
Others	1.361	1.200–1.544	<0.001*	1.213	1.064–1.382	0.004*
**Grade**						
Well/Moderate	1			1		
Poor/Undifferentiated	1.347	1.235–1.469	<0.001*	1.255	1.146–1.374	<0.001*
Unknown	0.807	0.657–0.990	0.040*	0.801	0.651–0.985	0.036*
**No. of resected lymph nodes**						
0	1			1		
1–3	0.841	0.685–1.034	0.100	0.785	0.635–0.970	0.025*
≥4	0.645	0.533–0.780	<0.001*	0.587	0.481–0.717	<0.001*
Unknown	0.706	0.543–0.919	0.009*	0.654	0.500–0.855	0.002*
**Tumor size (mm)**						
≤ 20	1			1		
21–30	1.398	1.230–1.589	<0.001*	1.316	1.157–1.496	<0.001*
31–40	1.432	1.279–1.603	<0.001*	1.310	1.167–1.470	<0.001*
**History of malignancy**						
No				1		
Yes	0.332	0.299–0.368	<0.001*	0.311	0.280–0.345	<0.001*
**Surgical procedure**						
Lobectomy	1			1		
Segmentectomy	1.224	1.017–1.473	0.033*	1.172	0.963–1.427	0.114

* *indicates that the difference was statistically significant*.

### Subgroup Analysis

To further explore the impact of TS on the choice of surgical strategy for stage IB NSCLC patients, subgroup analyses were conducted. As shown in Table 4, multivariate Cox regression analysis showed that segmentectomy yielded similar OS to that of lobectomy for NSCLC patients with TS ≤ 30 mm [TS ≤ 20 mm: HR = 1.068, 95% CI (0.853, 1.336), *P* = 0.566; TS > 20 and ≤ 30 mm: HR = 1.195, 95% CI (0.961, 1.487), *P* = 0.109], while in NSCLC patients with a TS of ≤ 40 and >30 mm, segmentectomy was associated with poorer OS [HR = 1.278, 95% CI (1.075, 1.520), *P* = 0.006]. We should note that in T2aN0M0 stage patients, if the size of tumor was ≤ 30 mm, the visceral pleural must have been invaded. As depicted in Table 5, segmentectomy achieved similar LCSS to that of lobectomy [TS ≤ 20 mm: HR = 1.029, 95% CI (0.682, 1.552), *P* = 0.893; TS > 20 and ≤ 30 mm, HR = 1.144, 95% CI (0.795, 1.645), *P* = 0.469]. However, segmentectomy yielded worse LCSS [HR = 1.118, 95% CI (1.005, 1.280), *P* = 0.047] in NSCLC patients with a TS of ≤ 40 and >30 mm.

**Table 4:** Subgroup analyses stratified by tumor size for overall survival.

	**TS** **≤** **20 mm**			**20 mm** **<** **TS** **≤** **30 mm**			**30 mm** **<** **TS** **≤** **40 mm**		
	**HR**	**95% CI**	***P***	**HR**	**95% CI**	**P**	**HR**	**95% CI**	***P***
**Surgical procedures**									
Lobectomy	1			1			1		
Segmentectomy	1.068	0.853–1.336	0.566	1.195	0.961–1.487	0.109	1.278	1.075–1.520	0.006*
**Age**									
≤ 60	1			1			1		
61–75	1.509	1.271–1.792	<0.001*	1.555	1.306–1.851	<0.001*	1.594	1.412–1.800	<0.001*
>75	2.179	1.802–2.635	<0.001*	2.431	2.031–2.910	<0.001*	2.516	2.221–2.849	<0.001*
**Grade**									
Well/Moderate	1			1			1		
Poor/Undifferentiated	1.118	0.977–1.279	0.103	1.180	1.050–1.325	0.005*	1.198	1.111–1.291	<0.001*
Unknown	0.914	0.694–1.203	0.520	0.880	0.682–1.136	0.327	0.838	0.710–0.990	0.038*

* *indicates that the difference was statistically significant*.

*TS, tumor size*.

**Table 5:** Subgroup analyses stratified by tumor size for lung cancer specific survival.

	**TS** **≤** **20 mm**			**20 mm** **<** **TS** **≤** **30 mm**			**30 mm** **<** **TS** **≤** **40 mm**		
	**HR**	**95% CI**	**P**	**HR**	**95% CI**	**P**	**HR**	**95% CI**	**P**
**Surgical procedure**									
Lobectomy	1			1			1		
Segmentectomy	1.029	0.682–1.552	0.893	1.144	0.795–1.645	0.469	1.118	1.005–1.280	0.047*
**Age**									
≤ 60	1			1			1		
61–75	1.317	1.026–1.691	0.031*	1.283	1.009–1.631	0.042*	1.485	1.251–1.764	<0.001*
>75	1.376	1.010–1.874	0.043*	1.607	1.242–2.81	<0.001*	1.805	1.503–2.167	<0.001*
**Grade**									
Well/Moderate	1			1			1		
Poor/Undifferentiated	1.266	1.012–1.583	0.039*	1.324	1.103–1.589	0.003*	1.231	1.092–1.387	0.001*
Unknown	0.902	0.562–1.450	0.671	0.970	0.659–1.428	0.878	0.698	0.523–0.933	0.015*

* *indicates that the difference was statistically significant*.

*TS, tumor size*.

## Discussion

Although lobectomy is recognized as the standard surgical treatment for patients with stage I NSCLC (13), the optimal surgical management of early-stage lung cancer still remains controversial. In recent years, with the development of surgical technological devices, surgical skills, and perioperative care, minimally invasive surgery has been increasingly accepted. More and more attention has been focused on sublobar resection as an alternative treatment for patients with early-stage NSCLC, especially the elderly ones and those with slight or moderate impairment of lung function. Moreover, patients who had undergone limited resection are more tolerant of reoperation if lung malignancies recurred. In our study, we observed that lobectomy achieved better OS and LCSS than did segmentectomy; however, after being adjusted by other prognostic factors, no significant difference was observed in LCSS between them. To the best of our knowledge, this is the first study to compare the survival rates after lobectomy and segmentectomy for the eighth edition of stage IB NSCLC patients.

In 1995, a study by Ginsberg et al. (13) demonstrated that lobectomy should be recommended as the standard treatment for patients with peripheral T1N0 non-small-cell lung cancer. However, recent studies have tried to assess the difference in outcomes between lobectomy and sublobar resection. Wolf and his team members found that lobectomy was associated with longer OS and recurrence-free survival in NSCLC patients with TS ≤ 2 cm (14). However, other studies (6, 15–18) reported that segmentectomy was comparable to lobectomy in terms of OS for stage IA NSCLC. There was also evidence from several studies of meta-analysis suggesting that segmentectomy could achieve comparable OS to lobectomy for stage IA NSCLC (19–21). It seemed that more and more studies suggested that segmentectomy could be the optimal surgical treatment for patients with stage IA NSCLC.

Numerous published studies explored the impact of segmentectomy and lobectomy on the prognosis of patients with stage IA or I NSCLC. It should be noted that stage I contains stages IA and IB. Most of previous studies of lung cancer were based on the seventh or sixth edition. T2N0M0 (T > 30 mm) was defined as stage IB in the sixth edition, and T2aN0M0 (T > 30 mm and T ≤ 50 mm) was defined as stage IB in the seventh. In 2012, Schuchert et al. (22) found that patients with stage IB NSCLC who underwent segmentectomy had reduced recurrence-free survival compared to those who underwent lobectomy. However, the TNM stage of the enrolled patients was classified according to the sixth edition of the American Joint Committee on Cancer (AJCC) Lung Cancer Staging System, and the outcomes of stage IB NSCLC patients would probably be different because of tumor staging based on different editions. Recently, Zhang et al. (23) reported that sublobar resection achieved an equivalent outcome to that of lobectomy, and similar results were also observed in propensity-matched analysis. But we should note that patients who underwent segmentectomy and wedge resection were all included in their study as sublobar resection. Segmentectomy belongs to anatomical resection, while wedge resection does not. Whitson et al. (24) suggested that lobectomy confers a significant survival advantage over segmentectomy in stage I NSCLC patients. However, the tumor staging system was based on the sixth edition lung cancer stage classification of the AJCC. Both the sixth and seventh editions are different from the newest eighth edition in IB NSCLC tumor staging (25). Obviously, these results are unfit to guide clinical strategies for stage IB NSCLC nowadays. Liu et al. (8) pointed out that NSCLC patients in stage T12N0M0 treated with lobectomy had a better outcome than those treated with sublobar resection. T12N0M0 stage contains stages IA, IB, and IIA. Moreover, sublobar resection contains segmentectomy and wedge resection. Therefore, we considered that the study by Liu et al. has limited directive significance to clinical strategies for patients with stage IB NSCLC. Our study included the patients with stage IB NSCLC according to the eighth edition lung cancer stage classification of the Union for International Cancer Control (UICC), of whom 557 received segmentectomy and 10,453 lobectomy. Due to the nature of retrospective study, multivariate Cox regression analyses were performed to remove the bias. The multivariate analyses showed that an older age, male sex, squamous cell carcinoma, advanced grade, a smaller numbers of resected lymph nodes, and a larger TS were independent prognostic factors, and they predicted worse OS and LCSS. Interestingly, patients with a history of malignancy had longer LCSS and seemed to have worse OS. However, the difference in OS was not significant between patients with such a history and those without. We speculated that the reason may be that patients with other malignancies were more likely to die of other recurrent cancers or long-term post-operative complications.

A previous study reported that mediastinal lymph node metastasis was significantly associated with poorer tumor differentiation degree, and a larger number of positive lymph nodes were significantly associated with worse OS and progression-free survival (26). Cao et al. (27) found that the more extensive the regional lymph node dissection was, the better the survival of patients who undergo sublobar resection for stage IA NSCLC with TS ≤ 2 cm. Our study also strongly demonstrated that a larger number of regional lymph nodes with dissection were associated with better LCSS and OS in stage IB patients. We speculated that the smaller the number of resected lymph nodes, the more likely the potential positive lymph nodes were unremoved. The potential unremoved positive lymph nodes may lead to the recurrence of lung cancer or distant metastasis after operation. Therefore, a thoracic surgeon should attach enough importance to the enlarged lymph nodes on CT/PET-CT before operation and remove the visible regional lymph nodes as much as possible.

In subgroups, multivariate analyses showed that segmentectomy yielded similar OS and LCSS for NSCLC patients with TS ≤ 30 mm compared with lobectomy. However, segmentectomy yielded worse OS and LCSS for NSCLC patients with TS > 30 mm and T ≤ 40 mm. We acknowledged that segmentectomy was likely to be performed in older patients who had a smaller TS, especially in those with an impaired lung function. In addition, more regional lymph nodes were likely to be resected when lobectomy was performed compared to segmentectomy. As discussed in the previous paragraph, the more the regional lymph nodes were resected, the better the outcome the patients would have. Taking the above factors into consideration, segmentectomy may be acceptable for appropriately selected stage IB NSCLC patients with an older age and a smaller TS, especially for those with comorbidities. However, the conclusion needs to be validated by multicenter randomized controlled trials (RCTs).

Previous studies reported that segmentectomy was associated with less blood loss, shorter operation time, shorter chest drainage, and shorter hospital stay compared with lobectomy (23, 28). However, other studies suggested that there were no significant differences in post-operative mortality, overall complications, and prolonged air leakage rates between segmentectomy and lobectomy in stage IA NSCLC patients (29, 30). There was also high-quality evidence from the Cancer and Lymphoma Group (CALBG 140503) revealing that no significant differences were observed in post-operative complications in segmentectomy and lobectomy cohorts, except that segmentectomy was associated with a higher rate of air leakage (31). Several reasons may account for these conflicting results, such as differences in surgical technology, perioperative patient care, study population, and study design. Because of lack of information about blood loss, operation time, chest drainage, and hospital stay in SEER database, we could not further evaluate the difference in intraoperative and post-operative complications between segmentectomy and lobectomy. The difference in complications between segmentectomy and lobectomy needs to be further confirmed in large multicenter RCTs.

Additionally, there are some limitations in the study. First, recently, various kinds of targeted therapies and immunotherapies for lung adenocarcinoma have increasingly been applied. The outcome of these patients who received targeted therapy or immunotherapy may greatly differ from those who did not. Due to the lack of detailed data, the impacts of different targeted therapies and immunotherapy on OS and LCSS in patients with segmentectomy and lobectomy could not be further assessed. Nevertheless, early-stage NSCLC patients were less likely to receive such treatment. Therefore, our conclusion may not have been substantially affected. Second, because of the nature of a retrospective study, some bias was inevitable. Our results need to be further validated by a larger randomized study cohort in the future. Finally, no detailed data about the positive rate of resected regional lymph nodes and surgical approach (open vs. VATS) were available, making the investigation further limited.

In conclusion, segmentectomy achieved equivalent OS and LCSS for stage IB NSCLC patients with TS ≤ 30 mm compared with lobectomy. Lobectomy yielded longer survival for IB NSCLC patients with TS > 30 mm and TS ≤ 40 mm. Therefore, segmentectomy may be acceptable for stage IB patients with an older age and a smaller TS, especially for those with impaired lung function.

## Data Availability Statement

All datasets generated for this study are included in the article/supplementary material.

## Author Contributions

BH, LZ, and TF: study design, manuscript writing, and final approval. BL and WJ: Data collection and analysis. HH and QG: Manuscript revision and final approval.

## Conflict of Interest

The authors declare that the research was conducted in the absence of any commercial or financial relationships that could be construed as a potential conflict of interest.
